# Supplementary material for: Nitric oxide controls shoot meristem activity via regulation of DNA methylation
Source: Nat Commun. 2023 Dec 4;14:8001. doi: 10.1038/s41467-023-43705-1 (PMC10696095; doi:10.1038/s41467-023-43705-1)
Supplement: Supplementary file 3 — Description of Additional Supplementary Files [file 41467_2023_43705_MOESM3_ESM.pdf]

Description of Additional Supplementary Files

Supplementary Data 1 List of Up-regulated genes in the shoot apex transcriptome of ap1/cal treated with 1mM SNP.

Supplementary Data 2 List of Down-regulated genes in the shoot apex transcriptome of ap1/cal treated with 1mM SNP.

Supplementary Data 3 List of *WUS/CLV3* domain enriched genes and S-nitrosylated proteins.

Supplementary Data 4 List of co-targets of AGO4 and WUS.

Supplementary Data 5 List of DMRs.

Supplementary Data 6 DNA constructs generated in this study.

Supplementary Data 7 Oligonucleotides used in this study (5'→3').
