# Supplementary material for: Nitric oxide controls shoot meristem activity via regulation of DNA methylation
Source: Nat Commun. 2023 Dec 4;14:8001. doi: 10.1038/s41467-023-43705-1 (PMC10696095; doi:10.1038/s41467-023-43705-1)
Supplement: Supplementary file 5 — Reporting Summary [file 41467_2023_43705_MOESM5_ESM.pdf]

nature portfolio

Corresponding author(s): Jan U. Lohmann

Last updated by author(s): Nov 6, 2023

Reporting Summary

Nature Portfolio wishes to improve the reproducibility of the work that we publish. This form provides structure for consistency and transparency in reporting. For further information on Nature Portfolio policies, see our Editorial Policies and the Editorial Policy Checklist.

Statistics

For all statistical analyses, confirm that the following items are present in the figure legend, table legend, main text, or Methods section.

n/a	Confirmed	
☐	☒	The exact sample size (*n*) for each experimental group/condition, given as a discrete number and unit of measurement
☐	☒	A statement on whether measurements were taken from distinct samples or whether the same sample was measured repeatedly
☐	☒	The statistical test(s) used AND whether they are one- or two-sided<br>*Only common tests should be described solely by name; describe more complex techniques in the Methods section.*
☒	☐	A description of all covariates tested
☐	☒	A description of any assumptions or corrections, such as tests of normality and adjustment for multiple comparisons
☐	☒	A full description of the statistical parameters including central tendency (e.g. means) or other basic estimates (e.g. regression coefficient) AND variation (e.g. standard deviation) or associated estimates of uncertainty (e.g. confidence intervals)
☐	☒	For null hypothesis testing, the test statistic (e.g. *F*, *t*, *r*) with confidence intervals, effect sizes, degrees of freedom and *P* value noted<br>*Give P values as exact values whenever suitable.*
☒	☐	For Bayesian analysis, information on the choice of priors and Markov chain Monte Carlo settings
☒	☐	For hierarchical and complex designs, identification of the appropriate level for tests and full reporting of outcomes
☒	☐	Estimates of effect sizes (e.g. Cohen's *d*, Pearson's *r*), indicating how they were calculated

Our web collection on statistics for biologists contains articles on many of the points above.

Software and code

Policy information about availability of computer code

Data collection	The following softwares were used to collect data.<br>High-throughput sequencing was performed with Illumina platform.<br>Microscopy images were collected by an upright Nikon A1 Confocal with a CFI Apo LWD 25×water immersion objective (Nikon Instruments).
Data analysis	The following publicly available software were used to analyze data.<br>R package "DEGseq" (DESeq2 version 3.11) was used for calculating the differentially expressed genes.<br>ImageJ (Fiji) was used for the measurement of meristem.<br>Heatmaps were generated using the “heatmap” or “heatmap2” program in R project, version 3.1.0.<br>Differences between groups were identified using Student’s t-test, and the p-value level was set at 5%.

For manuscripts utilizing custom algorithms or software that are central to the research but not yet described in published literature, software must be made available to editors and reviewers. We strongly encourage code deposition in a community repository (e.g. GitHub). See the Nature Portfolio guidelines for submitting code & software for further information.

Data

Policy information about availability of data

All manuscripts must include a data availability statement. This statement should provide the following information, where applicable:

- Accession codes, unique identifiers, or web links for publicly available datasets
- A description of any restrictions on data availability
- For clinical datasets or third party data, please ensure that the statement adheres to our policy

Sequence data sets generated during the current study are available in the NCBI Gene Expression Omnibus (GEO) and accessible through GEO series accession numbers GSE216952 (https://www.ncbi.nlm.nih.gov/geo/query/acc.cgi?acc=GSE216952) and GSE243447 (https://www.ncbi.nlm.nih.gov/geo/query/acc.cgi?acc=GSE243447).

Research involving human participants, their data, or biological material

Policy information about studies with human participants or human data. See also policy information about sex, gender (identity/presentation), and sexual orientation and race, ethnicity and racism.

Reporting on sex and gender	does not apply
Reporting on race, ethnicity, or other socially relevant groupings	does not apply
Population characteristics	does not apply
Recruitment	does not apply
Ethics oversight	does not apply

Note that full information on the approval of the study protocol must also be provided in the manuscript.

Field-specific reporting

Please select the one below that is the best fit for your research. If you are not sure, read the appropriate sections before making your selection.

[x] Life sciences   [ ] Behavioural & social sciences   [ ] Ecological, evolutionary & environmental sciences

For a reference copy of the document with all sections, see nature.com/documents/nr-reporting-summary-flat.pdf

Life sciences study design

All studies must disclose on these points even when the disclosure is negative.

Sample size	All the samples for mRNA expression, protein abundance, ChIP, EMSA, SAM size measurement and in situ hybridization experiments were treated the same way. We decided the sample sizes based on our knowledge acquired from our previous experience and from those used by other laboratories in similar studies. Sample sizes are indicated in the main text, Figures and legends. We confirmed that the sample sizes that we used in this study were adequate by performing biological replicates and obtaining similar results.
Data exclusions	No data were excluded from the analyses.
Replication	All experiments have been independently replicated at least twice in this study.
Randomization	All samples were exposed to similar growth conditions and control, and without any selection criteria. And large populations of genetically individuals were taken to minimize bias. For instance, In qRT-PCR, samples were randomly allocated to 3 groups as 3 independent biological replicates.
Blinding	In all independent experiment, all groups were analyzing together without data or sample exclusion. Yes, we were blinded to group allocation during data collection and analysis.

Reporting for specific materials, systems and methods

We require information from authors about some types of materials, experimental systems and methods used in many studies. Here, indicate whether each material, system or method listed is relevant to your study. If you are not sure if a list item applies to your research, read the appropriate section before selecting a response.

Materials & experimental systems

n/a	Involved in the study
☐	☒ Antibodies
☒	☐ Eukaryotic cell lines
☒	☐ Palaeontology and archaeology
☒	☐ Animals and other organisms
☒	☐ Clinical data
☒	☐ Dual use research of concern
☐	☒ Plants

Methods

n/a	Involved in the study
☐	☒ ChIP-seq
☒	☐ Flow cytometry
☒	☐ MRI-based neuroimaging

Antibodies

Antibodies used

The GFP antibody (supplier: Abcam, catalog number: ab290), the anti-DIG-AP (supplier: Roche, Cat. #11093274910), the mCherry antibody (supplier: Abcam, catalog number: ab125096), the AGO4 antibody (Agrisera, AS09617) and the GR antibody (supplier: Santa Cruz, sc-393232). The dilution rates are 1:500 for GFP antibody, 1:1250 for anti-DIG-AP, 1:500 for mCherry antibody, 1:1000 for AGO4 antibody and 1:200 for GR antibody.

Validation

Validations are based on the datasheet from the manufacturer and can found the detail information in the following list.
GFP antibody (https://www.abcam.com/products/primary-antibodies/gfp-antibody-ab290.html)
anti-DIG-AP (https://www.sigmaaldrich.com/catalog/product/roche/11093274910?lang=en&region=AU)
mCherry antibody (https://www.abcam.com/products/primary-antibodies/mcherry-antibody-1c51-ab125096.html)
GR antibody (https://www.scbt.com/p/gr-antibody-g-5)
AGO4 antibody (https://www.agrisera.com/en/artiklar/ago4-argonaute-4.html)

Dual use research of concern

Policy information about dual use research of concern

Hazards

Could the accidental, deliberate or reckless misuse of agents or technologies generated in the work, or the application of information presented in the manuscript, pose a threat to:

No	Yes
☒	☐ Public health
☒	☐ National security
☒	☐ Crops and/or livestock
☒	☐ Ecosystems
☒	☐ Any other significant area

Experiments of concern

Does the work involve any of these experiments of concern:

No	Yes
☒	☐ Demonstrate how to render a vaccine ineffective
☒	☐ Confer resistance to therapeutically useful antibiotics or antiviral agents
☒	☐ Enhance the virulence of a pathogen or render a nonpathogen virulent
☒	☐ Increase transmissibility of a pathogen
☒	☐ Alter the host range of a pathogen
☒	☐ Enable evasion of diagnostic/detection modalities
☒	☐ Enable the weaponization of a biological agent or toxin
☒	☐ Any other potentially harmful combination of experiments and agents

Plants

Seed stocks

Arabidopsis thaliana Col-0 was used as genetic background throughout the study with the exception of wus-7, which is in the Ler background. Seeds for mutants and wild type were obtained from NASC or individual labs, as noted in the manuscript.

Novel plant genotypes	-
Authentication	PCR based genotyping was used to validate genotypes.

ChIP-seq

Data deposition

- [x] Confirm that both raw and final processed data have been deposited in a public database such as GEO.
- [x] Confirm that you have deposited or provided access to graph files (e.g. BED files) for the called peaks.

Data access links<br>*May remain private before publication.*	GEO: GSE216952 (https://www.ncbi.nlm.nih.gov/geo/query/acc.cgi?acc=GSE216952)
Files in database submission	UBQ10::mCherry-GR-WUS, eChIP Rep1, 329IP1.<br>UBQ10::mCherry-GR-WUS, eChIP Rep2, 329IP2.<br>UBQ10::mCherry-GR-WUS,input Rep1, 329Input1.<br>UBQ10::mCherry-GR-WUS,input Rep2, 329Input2.<br>UBQ10::mCherry-GR,eChIP Rep1, 394IP1.<br>UBQ10::mCherry-GR,Input Rep1, 394Input1.
Genome browser session<br>(e.g. UCSC)	*Provide a link to an anonymized genome browser session for "Initial submission" and "Revised version" documents only, to enable peer review. Write "no longer applicable" for "Final submission" documents.*

Methodology

Replicates	Two replicates for UBQ10::mCherry-GR-WUS and one replicate for UBQ10::mCherry-GR
Sequencing depth	Read length 75bp, single-end<br>UBQ10::mCherry-GR-WUS, eChIP Rep1, 64377038 reads.<br>UBQ10::mCherry-GR-WUS, eChIP Rep2, 54788065 reads.<br>UBQ10::mCherry-GR-WUS,input Rep1, 129450362 reads.<br>UBQ10::mCherry-GR-WUS,input Rep2, 136424817 reads.<br>UBQ10::mCherry-GR,eChIP Rep1, 51797608 reads.<br>UBQ10::mCherry-GR,Input Rep1, 143290748 reads.
Antibodies	RFP-Trap Magnetic Agarose (ChromoTek, rtma-20)
Peak calling parameters	ChIP peak-calling was performed using MACS2 (Zhang Y, Liu T, Meyer CA, Eeckhoute J, Johnson DS, Bernstein BE, Nusbaum C, Myers RM, Brown M, Li W, Liu XS. (2008) Model-based Analysis of ChIP-Seq (MACS), Genome Biology, 2008;9(9):R137.) using following parameters: -g 1.19e+8, -q 0.01
Data quality	To ensure that our data were of high quality, we kept only called peaks with posterior probability higher that 0.9.
Software	ChIP-seq data were mapped to TAIR10 genome by BWA aligner.<br>Peak calling was performed using MACS2.
